# Biomaterials-Mediated Tumor Infarction Therapy

**DOI:** 10.3389/fbioe.2022.916926

**Published:** 2022-06-09

**Authors:** Shizheng Tong, Wei Zhao, Duoyi Zhao, Weilin Zhang, Zhiyu Zhang

**Affiliations:** Department of Orthopedics, The Fourth Affiliated Hospital of China Medical University, Shenyang, China

**Keywords:** infarction, tumor vessels, biomaterial, cancer therapy, peptide

## Abstract

Agents for tumor vascular infarction are recently developed therapeutic agents for the vascular destruction of tumors. They can suppress the progression of the tumor by preventing the flow of nutrition and oxygen to its tissues. Agents of tumor vascular infarction can be divided into three categories according to the differences in their pathways of action: those that use the thrombin-activating pathway, fibrin-activating pathway, and platelet-activating pathway. However, poor targeting ability, low permeation, and potential side-effects restrict the development of the corresponding drugs. Biomaterials can subtly avoid these drawbacks to suppress the tumor. In this article, the authors summarize currently used biomaterials for tumor infarction therapy with the goal of identifying its mechanism, and discuss outstanding deficiencies in methods of this kind.

## Introduction

Tumor vascular infarction-based therapy was proposed by Huang et al. to treat vascular-rich tumors (Huang et al., 1997). And vascular blockade therapy and anti-angiogenic therapy together form a new and popular direction of oncology treatment - tumour vascular blockade therapy. Note that both vascular infraction and anti-angiogenesis approaches cut down the nutrients and oxygen supply to the tumor, which ultimately starve the cancer cells. Anti-angiogenesis destruct the neovasculature formation (Oba et al., 2010; Dirisala et al., 2014). In contrast, vascular infraction approaches rely on clot formation within the tumor blood vessels to occlude the blood vessels that feed the tumor, subsequently causing necrosis and apoptosis of neoplastic cells. The effect of infarction treatment is almost immediate, in contrast to chemotherapy (Pizzamiglio et al., 2021). For example, breast cancer needs to be treated with trastuzumab for at least 9 weeks, whereas infarction treatment requires only about 1/10 of this time (Baselga et al., 1996). Moreover, infarction treatment does not involve directly attacking the tumor cells or influencing their pathways, and thus is unlikely to lead to the development of resistance during treatment. The distribution of disordered blood vessels and the hypoxic condition of the microenvironment of the tumor result in hypercoagulable blood, which provides favorable conditions for the formation of the thrombus (Tilki et al., 2007).

However, few infarction drugs are currently used in clinical practice. Only a truncated form of the tissue factor (tTF) has been approved by the U.S. Food And Drug Administration (FDA) (Huang et al., 1997; Zhu and Zhu, 2017). A few shortages have contributed to this situation. First, once blood-clotting substances form a thrombus in the normal tissues and block normal blood vessels, this causes secondary injuries to patients. Tumor patients in general have a high platelet count and high blood viscosity, which facilitates the formation of the thrombus to hinder the use of the agent (Hisada and Mackman, 2017; Haemmerle et al., 2018). Second, currently available clotting agents cannot completely block the blood vessels of the tumor, and the tumor cells can obtain nutrients from the remaining blood vessels or the normal surrounding environment (Berdel et al., 2021). Third, compared with normal blood vessels that have a complete morphology and distinct layers, tumorous blood vessels are primitive, usually lack smooth muscle cells and pericytes, and mainly rely on endothelial cells to transport nutrients and oxygen. The distribution of blood vessels in the tumor is thus disordered and the blood pressure is higher than normal, where this is not conducive to drug permeation (Flessner et al., 2005; Ding et al., 2019).

The above shows that treating tumors by using clotting drugs alone is a path riddled with obstacles. In this circumstance, the idea of combined therapy based on biomaterials and infarction agents offers promise to inhibit tumor progression (Zheng et al., 2020; Zhao D. et al., 2021; Chen et al., 2021). First, currently available biomaterials (such as peptides, nanorobots, and antibodies) functionalize therapeutic drugs, thus enabling the agent to precisely target endothelial cells of the tumor, improve the safety of the drug, and enhance its anti-tumor effects (Seidi et al., 2018; Li et al., 2020; Zhao Y. et al., 2021; Drzyzga et al., 2021). Second, biomaterials are used as carriers to simultaneously carry multiple therapeutic agents for combined treatment to damage tumor cells in a multi-dimensional and comprehensive manner (Colli et al., 2017; Wei et al., 2021; Zheng and Ding, 2022). Third, the biomaterials ensure that the therapeutic agent that may cause adverse reactions in normal tissues is slow-released, thereby providing a higher dose than if it were administered alone (Feng et al., 2020; Feng et al., 2021; Yang et al., 2021). Fourth, the materials prolong the duration of circulation of the drug in the blood (Jiang et al., 2019; Xue et al., 2020; An et al., 2021). Most therapeutic agents cannot exist in the blood for a long time (Pippa et al., 2020). For example, thrombin is metabolically deactivated within a few minutes under normal physiological conditions (Ding et al., 2019). However, the emergence of such biomaterials as nanorobots and mesoporous silica nanoparticles prolongs the duration of circulation of therapeutic agents in the blood (Xiong et al., 2018; Li et al., 2019; Berdel et al., 2020; Li et al., 2020). With this extended circulation, drugs that previously could not be used directly can accumulate on tissues of the tumor and kill the tumor cells.

Most clotting drugs cause vascular infarction through different clotting pathways: by activating the fibrin, platelets, and thrombin (Table 1). Coagulation involves the conversion of fibrinogen into fibrin as well as platelet activation and aggregation to form a hemostatic thrombus (Ruggeri and Mendolicchio, 2007; Kearney et al., 2021). There is a connection between the steps, whereby thrombin can activate fibrinogen and certain coagulation-related factors to stimulate platelet aggregation. When the body is functioning normally, the coagulation factors are cleared by macrophages to avoid coagulation (Majerus and Miletich, 1978; Ho-Tin-Noe et al., 2009; Ahnstrom and Gilbert, 2021). But in a majority of tumor patients, the composition of the blood represents a disorder that makes it susceptible to clots. This can not only activate the conversion of fibrinogen into fibrin through the coagulation pathway, but can also induce platelet aggregation.

**TABLE 1:** Summary of biomaterials for tumor infarction therapy.

Strategy	Biomaterial	Infarction Agents	Cancers	Refs
Activation of thrombin pathway	NGR	tTF	Human SCLC xenograft	Schmidt et al. (2017)
	AS1411		MHCC-97H, B16–F10	Li et al. (2021b)
	CREKA		4T1, MHCC97H, LS174T	Xiong et al. (2018)
	Anti-NRP-LmAb		HT1080	Kessler et al. (2018)
	EG3287 and *O*-carboxymethyl chitosan-coated iron oxide nanoparticles		HepG2	Zou et al. (2020)
	Nanorobot	Thrombin	MDA-MB231, B16-F10, SK-OV3	Li et al. (2018)
	Chitosan-based polymer nanoparticles		MDA-MB231, B16-F10, SK-OV3	Li et al. (2020)
Activation of fibrin pathway	NGR	Coagulase	4T1	Seidi et al. (2018)
	RGD		CT26, 4T1, and SKOV3	Jahanban-Esfahlan et al. (2017)
	Laminin mimic peptide	Fibrin	MDA-MB231	Zhang et al. (2020)
Activation of platelet pathway	SSRBC	Combretastatin A4	Humanized HbS-knockin	Sun et al. (2019)
	Platelet membrane-wrapped mesoporous silica nanoparticles	Combretastatin A4	MHCC-97H	Di Paolo et al. (2013)
	Platelet-like nanoparticles	—	MDA-MB-231	Yang et al. (2020)

**SCHEME 1:**
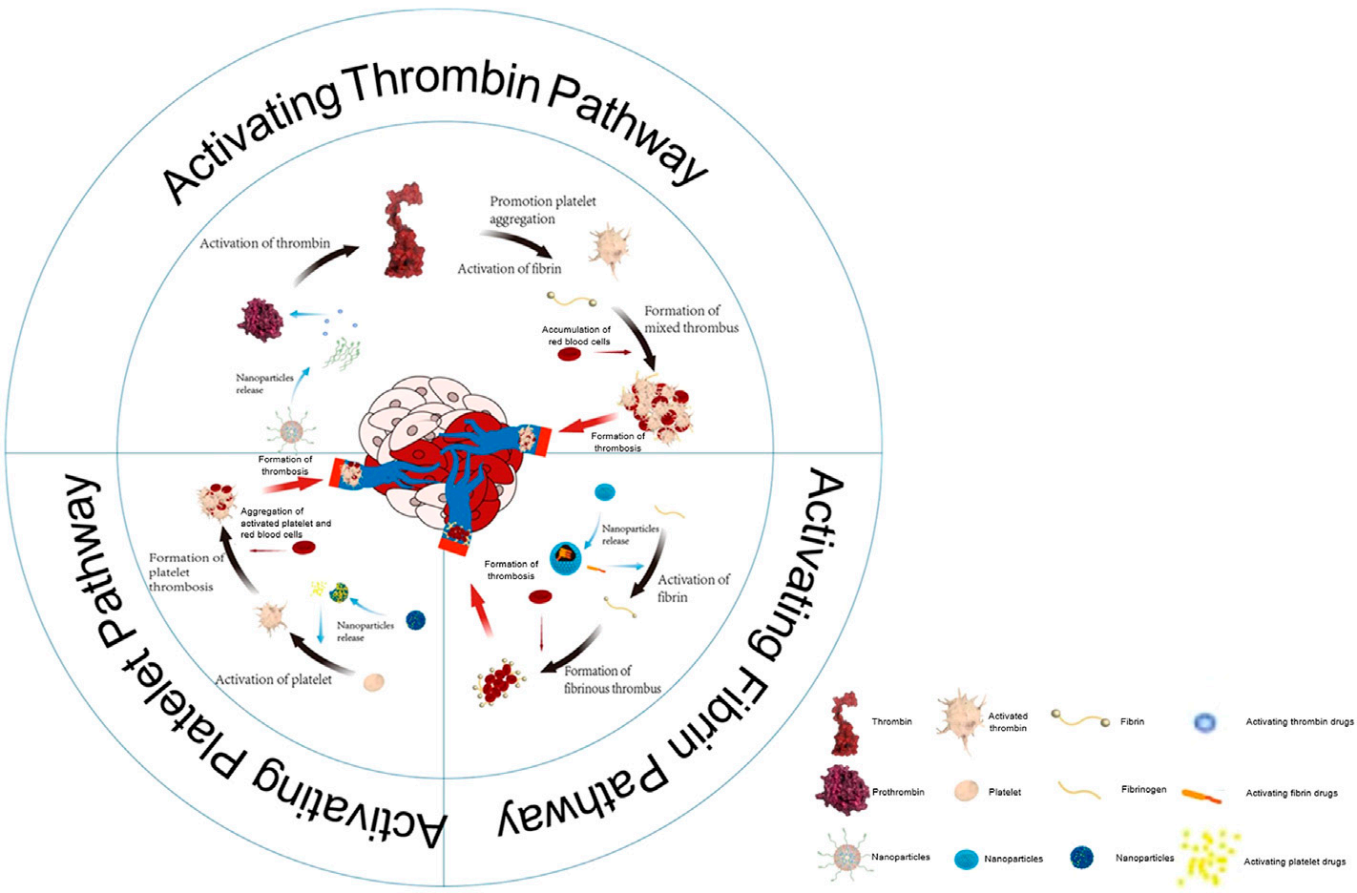
The vascular infarction treatment activates three pathways: the thrombin pathway, fibrin pathway, as well as platelet pathway.

Here, we divided the article into three parts according to the main activation direction of the treatment plan, as shown in Scheme 1. First, we consider the activation of the thrombin pathway. The classic drug in this context is tTF that, as the initiator of a pathway for exogenous clotting in the human body, is modified at the tumor site to produce clotting through biomaterials (Bieker et al., 2009; Schmidt et al., 2017; Xiong et al., 2018; Hoink et al., 2019; Xu et al., 2019; Berdel et al., 2020; Schliemann et al., 2020; Zou et al., 2020; Berdel et al., 2021). Second, we consider the activation of the fibrin pathway. In contrast to the pathway for exogenous coagulation, fibrinogen can be directly converted into fibrin to avoid the cascade reaction caused by the activation of a large number of coagulation-related factors (Jahanban-Esfahlan et al., 2017; Seidi et al., 2018; Daei Farshchi Adli et al., 2021). An abnormally large number of coagulation-related factors are likely to cause secondary harm to the patient. Third, we consider activating the platelet pathway. Biomaterials can deliver drugs that induce platelet aggregation at the sites of the tumor, or ones that destroy the vascular endothelial cells to expose the subvascular collagen, which in turn leads to platelet aggregation (Head and Jameson, 2010; Zweifel et al., 2011). We also discuss outstanding challenges faced by these strategies and directions of future research in the area.

## Biomaterials-Mediated Therapy Through Activation of Thrombin Pathway

The thrombin pathway causes thrombus mainly through the activation of thrombin to induce platelet aggregation and accelerate the conversion of fibrinogen to fibrin. The activated thrombin can provide positive feedback to expand the coagulation reaction as well as clotting substances for the pathway for exogenous coagulation (Van Der Meijden et al., 2012; Tripisciano et al., 2020).

The tTF containing the extracellular domain of the tissue factor has been used to induce thrombosis in tumorous blood vessels. It can cascade to the signaling pathways downstream to activate thrombin through pathways of exogenous clotting. The tTF has the following benefits: 1) Immune resistance. tTF derived from the human body has no activating effect on the immune system (Kessler et al., 2018; Luo et al., 2021). 2) High efficiency. The agents activate the relevant coagulation factors and quickly form thrombus through the pathway for exogenous coagulation (Fujikawa et al., 1974; Stern et al., 1985). However, the tTF cannot completely restrain tumor development. In addition, large doses of the tTF may affect normal tissues and organs. In this context, combining the tTF with other materials to reduce drug toxicity is a promising solution.

Schmidt et al. connected the NGR (asparagine-glycine-arginine)-targeting peptide to the end of the factor to modify the tTF to obtain tTF-NGR (Schmidt et al., 2017). This compound did not change the coagulation function of the tTF, whereas tTF-NGR had the ability to selectively target to tumor because NGR is a CD13-targeting peptide (Corti et al., 2008). tTF-NGR mainly targets the tTF with aminopeptidase N on angiogenic endothelial cells (Amin et al., 2018). It is the first candidate drug of this type for the coagulation ligand, and can be used on cancer patients in clinical research (Schliemann et al., 2020). And they established an HTB119 human small-cell lung cancer (SCLC) xenograft model, and injected tTF-NGR and saline once every 2 days for a total of six injections. At the end of the experiment, the tumor was removed for observation and comparison. Compared with the control group, the growth of the tumor in the tTF-NGR group was significantly reduced. The results also showed that the targeted tissue factor had a good inhibitory effect on the growth of SCLC tumors. Therefore, in subsequent clinical trials, Schliemann et al. used dose-increasing tTF-NGR treatment on 17 patients with advanced cancer who had exceeded standard therapies in a phase I study (Schliemann et al., 2020). The results again proved that tTF-NGR, as a drug for vascular therapy, can suppress the tumor and is safe.

**FIGURE 1:**
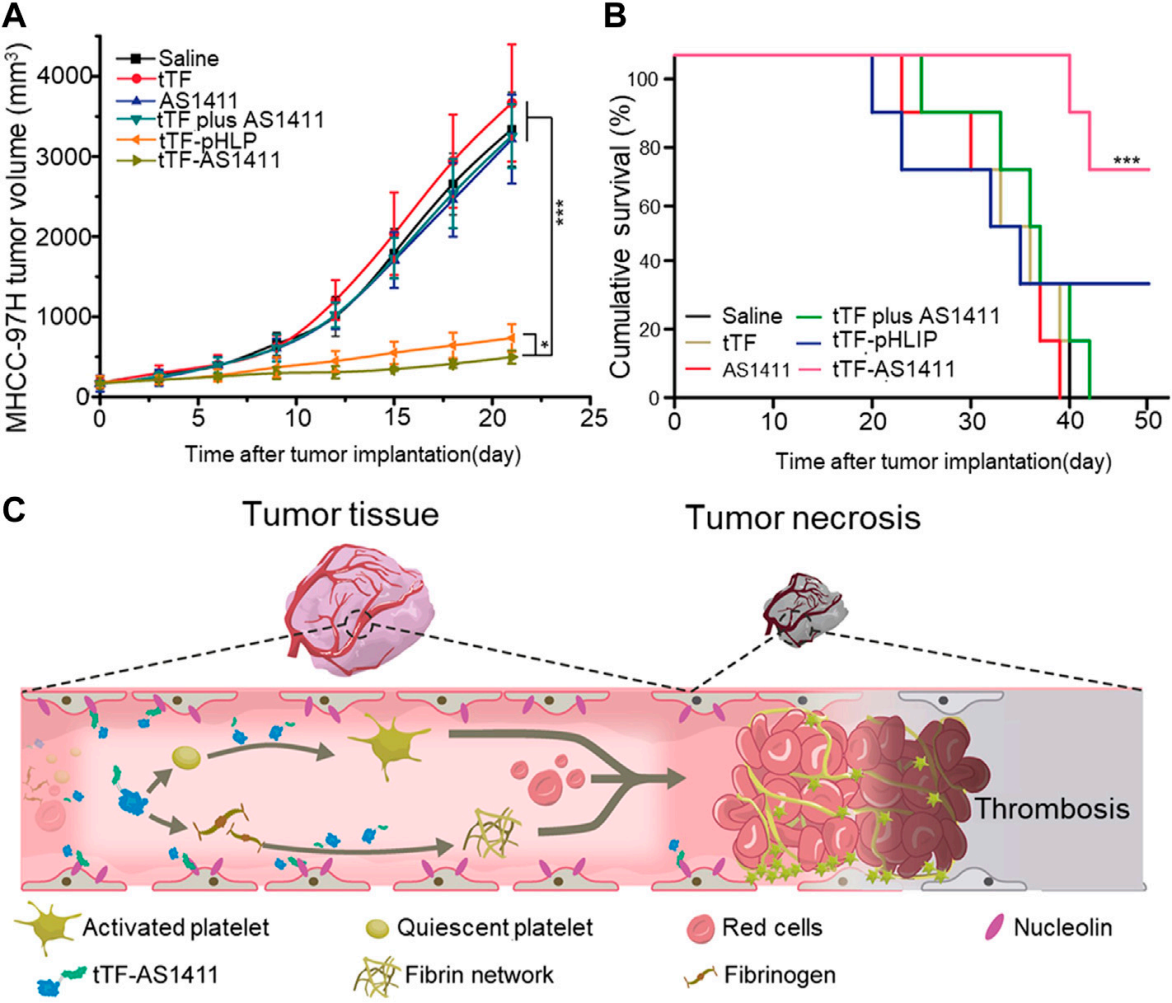
Antitumor activity of tTF-AS1411 **(A)** The inhibition of the growth of MHCC-97H liver tumors in mice **(B)** Cumulative survival of MHCC-97H tumor-bearing mice **(C)** Illustration of the hypothesized progression of tTF-AS1411 within tumor vessels. Reprinted with permission from (Li et al., 2021b).

However, the synthesis of the targeting peptide is complicated and costly, which hinders its large-scale application. Li et al. conjugated a DNA aptamer targeting nucleolin (a protein that is overexpressed in the liver tumors) to the tTF, through sulfosuccinimidyl 4-(*N*-maleimidomethyl) cyclohexane-1-carboxylate (sulfo-SMCC). The tTF-AS1411 conjugate could not only accurately target and inhibit the tumor, but also did not affect the normal tissues (Figure 1A) (Li et al., 2021b). The data showed that tTF-AS1411 could significantly inhibit the growth of liver tumors on metastasis of human hepatocellular carcinoma (MHCC-97H) (Figure 1B,C). Moreover, its simple process of preparation and low price enable the wide use of the conjugate. Nevertheless, AS1411 mainly acts on tumor cells rich in nucleolar protein, such as those that cause breast cancer, lung cancer, pancreatic cancer, and acute myeloid leukemia. The specificity of AS1411 decreases for tumor cells with low nucleolar protein expression.

Thus, a material that can target most tumors is needed. CREKA (Cys-Arg-Glu-Lys-Ala), as a tumor-homing peptide identified by phage display technology, can solve the above problem. It can recognize microthrombus-related fibrin–fibronectin complexes that are selectively overexpressed in the vascular endothelium and stroma of the tumor but not in normal tissues (Ruoslahti et al., 2010; Yamashita and Hashida, 2013).

Shi et al. established three types of tumor models—the 4T1 model, the MHCC97H liver tumor model, and the LS174T colon tumor model—and treated the tumors with tTF-CREKA. CREKA and tTF were optimized for the prokaryotic cells and purified with isopropyl-β-D-thiogalactoside (IPTG) for 4 hours to obtain tTF-CREKA (Xiong et al., 2018). The authors found that tTF-CREKA had an inhibitory effect on the tumors in the three models, especially in the 4T1 model, because this model featured more blood vessels than the other two (Pulaski and Ostrand-Rosenberg, 2001). The authors also compared tTF-CREKA with a truncated tissue factor with pH (low) insertion peptides (tTF-pHLIP). Under the same treatment conditions, the therapeutic effect of tTF-CREKA was almost the same as that of tTF-pHLIP on LS174T tumors but was more active against the 4T1 and MHCC97H tumors. This was mainly owing to its different effective positions on the tumor cells. Because pH-responsive drugs are not favorable for patients with diseases such as ischemic myocarditis (Estabragh and Mamas, 2013; Kociol et al., 2020), tTF-CREKA is more effective than tTF-pHLIP.

However, the specific site at which CREKA binds to tumor cells is relatively simple such that the tumor cells are prone to generate avoidance mechanisms to avoid binding to the polypeptides. Kessler et al. localized the anti-NRP-1-mAb to the surface of the vascular endothelial cells of the tumor to induce vascular infarction (16) (Kessler et al., 2018), through the coupling of water-soluble 1-ethyl-3-(3-dimethylaminopropyl) carbodiimide and *N*-hydroxysulfosuccinimide, to couple the mAb to the polypeptide. The anti-tumor activity of the mAb-SA:tTF-B system was evaluated in mice bearing HepG2,a hepatocellular carcinoma cell line. The growth of the tumor in the mice treated with mAb-SA:tTF-B decreased compared with the other treatment groups considered. However, the effect of coagulation of the material was too prominent, where this is not conducive to controlling the treatment dosage for patients, especially those with cardiovascular diseases. After all, the safety of the drug is considered the primary criterion for evaluating this strategy. Zou et al. subsequently proposed a fusion coagulant protein tTF-EG3287 comprising of the tTF and the NRP-1 targeting peptide EG3287and *O*-carboxymethyl chitosan-coated iron oxide nanoparticles be mixed as a magnetic carrier (Zou et al., 2020). tTF-EG3287 does not have a procoagulant effect on blood circulation. But when it is combined with neuropilin-1 (NRP-1), which is prominently expressed in tumor-related vascular endothelial cells, it yields a strong procoagulant capability (Min et al., 2019). The authors evaluated the anti-cancer activity of mesotetra(4-carboxyphenyl)porphyrin (mTCPP), a photosensitizer against HepG2 in tumor-bearing BALB/c nude mouse model, including in cases of subcutaneous transplantation and orthotopic transplantation. The results showed that after the intravenous injection of MTPCP, thrombosis in particular occurred in the tumor-related blood vessels. This retarded the growth of the tumor but did not damage the normal organs.

Thrombin is a key enzyme in the coagulation cascade that can cleave the plasma fibrinogen into fibrin monomers that can then spontaneously form insoluble polymers (Wells and Di Cera, 1992). Thrombin can also activate coagulation factors VIII (to VIIIa) and V (to Va) as well as platelets to aggregate platelets and block blood vessels (Downes et al., 2022). Of course, the role of thrombin is not limited to coagulation events. It also stimulates “mitosis” events by interacting with receptors on the cell surface, and plays a key role in the healing of wounds (Tao et al., 2021). Although thrombin has been used clinically as a therapeutic drug, the potential systemic side-effects caused by blood leakage during circulation, increase in values of the coagulation parameters, and avascular necrosis of normal tissues have hindered further research (Cines et al., 2014; Gajos et al., 2018). Therefore, a series of biomaterials that can enable thrombin to overcome these difficulties is in demand.

DNA origami technology was first applied to the transportation of substances in 2006, and greatly improved the feasibility of targeted drug therapy. Li et al. applied it to the treatment of vascular infarction (Li et al., 2018). The half-life of thrombin in the body is relatively short, which hinders its clinic use (Van Cott et al., 2017). To enable thrombin to persist for a longer time in the body, Li et al. combined it with a nanorobot to prevent the thrombin from being metabolized during transit to the target tumor cells. While the nanorobot was combined with the nucleolin in the tumor, it exposed the thrombin wrapped in the material to the surface. And when not bound to tumors, the nanorobots protected the thrombin from being cleared by macrophages as it circulated. The authors then compared two tumor models (of melanoma and ovarian cancer) to compare the effects of the material on different tumors based on differences in vascular density. Although the final results showed that the effect of treatment was much better on patients of melanoma than those of ovarian cancer, the nanomaterials were also found to have a significant inhibitory effect on ovarian cancer.Whereas, because the drug could not completely block the tumor’s supply of blood vessels, the tumor could still obtain the materials needed for growth (Schwoppe et al., 2013). Li et al. used an ion gel-based method to prepare chitosan-based polymer nanoparticles, and used it to integrate thrombin and the chemotherapy drug doxorubicin (Dox) into a single nanocarrier to kill tumors (Li et al., 2020). Unlike in case of the separate administration of the two drugs, this system produced a synergistic effect by simultaneously affecting two independent aspects of tumor viability. The results showed that the dual drug delivery system had a more powerful treatment effect than single drug delivery. When treating different tumor models, tumors with a large number of blood vessels have better therapeutic effects. In addition to being non-cytotoxic to normal tissues, this system has been proven to prolong the duration of circulation of the drug in the body and thus can better suppress the tumor.

## Biomaterials-Mediated Therapy Through Activation of Fibrin Pathway

The blood vessels of cancer patients are in an abnormal state, because of which the changes (of blood viscosity and haemodynamics) in blood brought about by the drug easily affect the human body. Moreover, a few coagulation factors stimulate the growth and metastasis of tumor cells (Cohen et al., 2018; Kalinich and Haber, 2018). These problems hinder the use of traditional blocking drugs, and this creates the need for a pathway to clotting that does not activate cascaded amplification. The literature has proposed that the activation of only fibrinogen to transform into fibrin, without activating the other coagulation substances, can minimize the impact of thrombus formation on the body (Zhang et al., 2020).

Coagulase is a substance similar to thrombin that can transform the fibrinogen in plasma into fibrin and coagulate the plasma (Tripisciano et al., 2020) (Friedrich et al., 2003; Luo et al., 2021). Because coagulase does not activate platelets nor change factors influencing blood coagulation, a growing number of researchers have attended to it. However, free coagulase has a short half-life in the body. Combining coagulase with biological materials to target tumorous blood vessels and extend the time of residence of the drugs in the body is now a trend in this field. Jahanban-Esfahlan et al. connected the truncated coagulase to RGD (Arg-Gly-Asp) (tCoa-RGD), where the coagulase retained its activity and had a close affinity with αvβ3 endothelial cell receptors (Jahanban-Esfahlan et al., 2017) (Bhagwat et al., 2001). To verify the effect of tCoa-RGD in terms of tumor inhibition, the authors established a CT26 mouse colon model, a 4T1 mouse breast model, and an SKOV3 human ovarian tumor model, and found that the systemic injection of lower doses of tCoa-RGD significantly inhibited solid tumor growth due to CT26, 4T1, and SKOV3 in animals. Thrombosis and a large number of necrotized tumor cells were also observed in the tumor tissues. The research showed that the tCoa-RGD fusion protein can induce thrombosis while ensuring non-toxicity in normal tissues.

**FIGURE 2:**
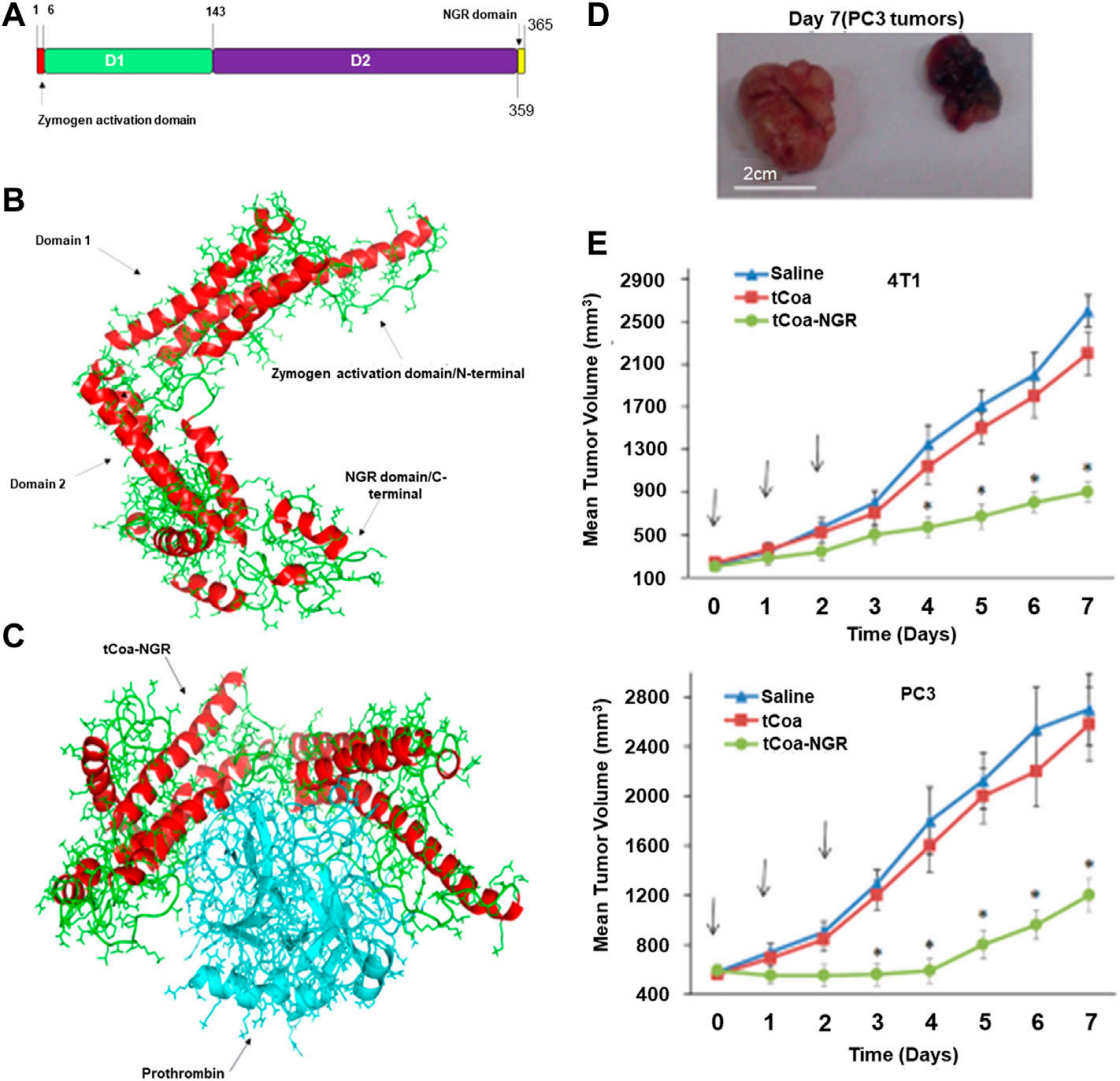
Molecular dynamic studies of tCoa-NGR proteins and therapeutic potential of tCoa-NGR proteins *in vivo* **(A)** Protein structure of tCoa-NGR **(B)** 3D structure of tCoa-NGR **(C)** Molecular dynamic simulation of tCoa-NGR-prothrombin complex **(D)**.Illustrative photos of mice bearing prostate cancer xenografts (PC3) at the end of treatment treated with tCoa-NGR (right) or tCoa (left) **(E)** 4T1 and PC3 tumor-bearing mice were treated by the indicated formulations. Reprinted with permission from (Seidi et al., 2018).

However, the RGD peptide recognizes only the αvβ3 integrin (Ghabraie et al., 2020), which are transmembrane heterodimers that protect against immune evasion. Seidi et al. used bi-specific NGR peptides that can recognize CD13 and αvβ3 to improve the efficiency of targeting of the drug and its tumor-killing ability (Figure 2A–C) (Seidi et al., 2018). The result of ELISA showed that when tTF-NGR and tTF-RGD were combined with HUVEC in a ratio of 1:10, the competitive inhibition due to tTF-NGR resulted in an 80% reduction in the binding capacity of tTF-RGD to HUVEC while tTF-NGR reduced this by only 46%. (Figure 2D,E) On the 4T1 breast cancer model of the mouse, tCoa-NGR showed a better effect of suppressing the tumor as well. However, due to the existence of the edge effect (Dienst et al., 2005), the remaining tumor cells continued to grow through blood vessels of the surrounding normal tissue and accelerated the metastasis of the cells. To solve this problem, Adli et al. combined the administration of vadimezan and recombinant coagulase-NGR to kill tumor cells (Daei Farshchi Adli et al., 2021). The volume of the tumor in mice in the melanoma model treated by the combined administration group was 73.12%, smaller than that of the control tumor. A combination of the two was not only effective for the infarction of tumorous blood vessels, but also changed the immune microenvironment around the cells, leading to changes in the concentration of cytokines and activating the immune cells, thus leading to immune responses (Krem and Di Cera, 2001; Gajewski et al., 2013).

In addition to using coagulase to activate fibrinogen to form a fibrin network, some researchers sent fibrin directly into tumorous blood vessels to mediate the vascular blockage of the tumor. This strategy was safer and more efficient than previous blocking methods. Zhang et al. designed a laminin mimetic peptide (LMMP), which has the hydrogen-bonding sequence Lys-Leu-Val-Phe-Phe (KLVFF) as part of fibrillation, to target the peptide sequence CREKA to bind the microthrombus, with the pH-responsive sequence His6 to modulate the speed of fibrillation and oligo-ethylene glycol to improve hydrophilicity. This biomaterial performed its function through the *in-situ* dual regulation of the pH values of tumorous blood vessels and the microthrombus. In the MDA-MB-231 tumor model, the authors compared the effects of LMMP-lacking CREKA, LMMP-lacking His6, and normal LMMP on tumor treatment, and found that they were equivalent with respect to a lack of a targeting function, a lack of pH responsiveness, and dually regulated drugs, respectively. The results showed that the dual regulation drugs groups had better targeting and tumor-suppressive effects than the others (Zhang et al., 2020).

## Biomaterials-Mediated Therapy Through Activation of Platelet Pathway

Platelet activation is commonly observed in cardiovascular diseases—for example, myocardial infarction and pulmonary embolism (Torbicki et al., 2008; Thygesen et al., 2019). The occurrence of the disease usually begins with injury to the endothelial cells, which leads to the exposure of the subcutaneous collagen. The exposed collagen (mainly type I and III collagen) then activates the platelets and provides adhesion sites for their aggregation to eventually form thrombus (Yuan et al., 1997; De Meyer et al., 2009; Smeets et al., 2017). Some researchers have tried to transfer this pathological process into the blood vessels of tumors. This strategy induces thrombus formation by artificially exposing the subcutaneous collagen by treating the vascular endothelial cells of the tumor with specific vascular destructors. Moreover, the environment of the tumor exhibits a state of high coagulation, which provides a substantial material basis for the implementation of this strategy.

However, currently commonly used drugs, such as combretastatin A4 and apatinib, have poor water solubility and low accuracy of targeting the tumor. CA4, as a clinical drug that has been marketed, has been approved by the FDA (Rastogi et al., 2018). It binds to tubulin in the cells to arrest cell division and induce apoptosis, especially in endothelial cells. It further hinders the sliding of microtubules and microfilaments, leading to the deformation of the structure of the endothelial cells, and attracts the neutrophils to provoke an immune response. Its efficient killing effect makes it unique in this field, but its low water solubility and cytotoxicity on normal organs limit its use. Most importantly, CA4, like other vessel infarction drugs, does not prevent the residual tumor cells from absorbing nutrients and oxygen from the surrounding normal tissue.

Solve the above problems is the focus of research in the area. Sun et al. combined the vascular disrupting agent combretastatin A-4 (CA-4) with sickle red blood cells (SSRBCs) to block blood vessels of the tumor (Sun et al., 2019). By combining SSRBCs, the intrinsic oxygen-sensing function of which allowed them to enter the hypoxic niche of the tumor, the agent induced tumor form micro-aggregates attracted platelet aggregation, and induced local blood vessel closure. *In vivo* test, the tumor volume of the combined treatment was smaller than that of the control group, because thrombosis formed in the tumor. However, the sickle-shaped red blood cells were large, and led to a high viscosity of blood flow (Byrnes and Wolberg, 2017; Sun et al., 2019). This problem cannot be ignored in patients with other vascular diseases (Bergan et al., 2006; Michel et al., 2012). In addition, the SSRBCs were able to activate the immune response, and were easily cleared by macrophages circulating in the blood (Rees et al., 2010; Gravitz and Pincock, 2014).

**FIGURE 3:**
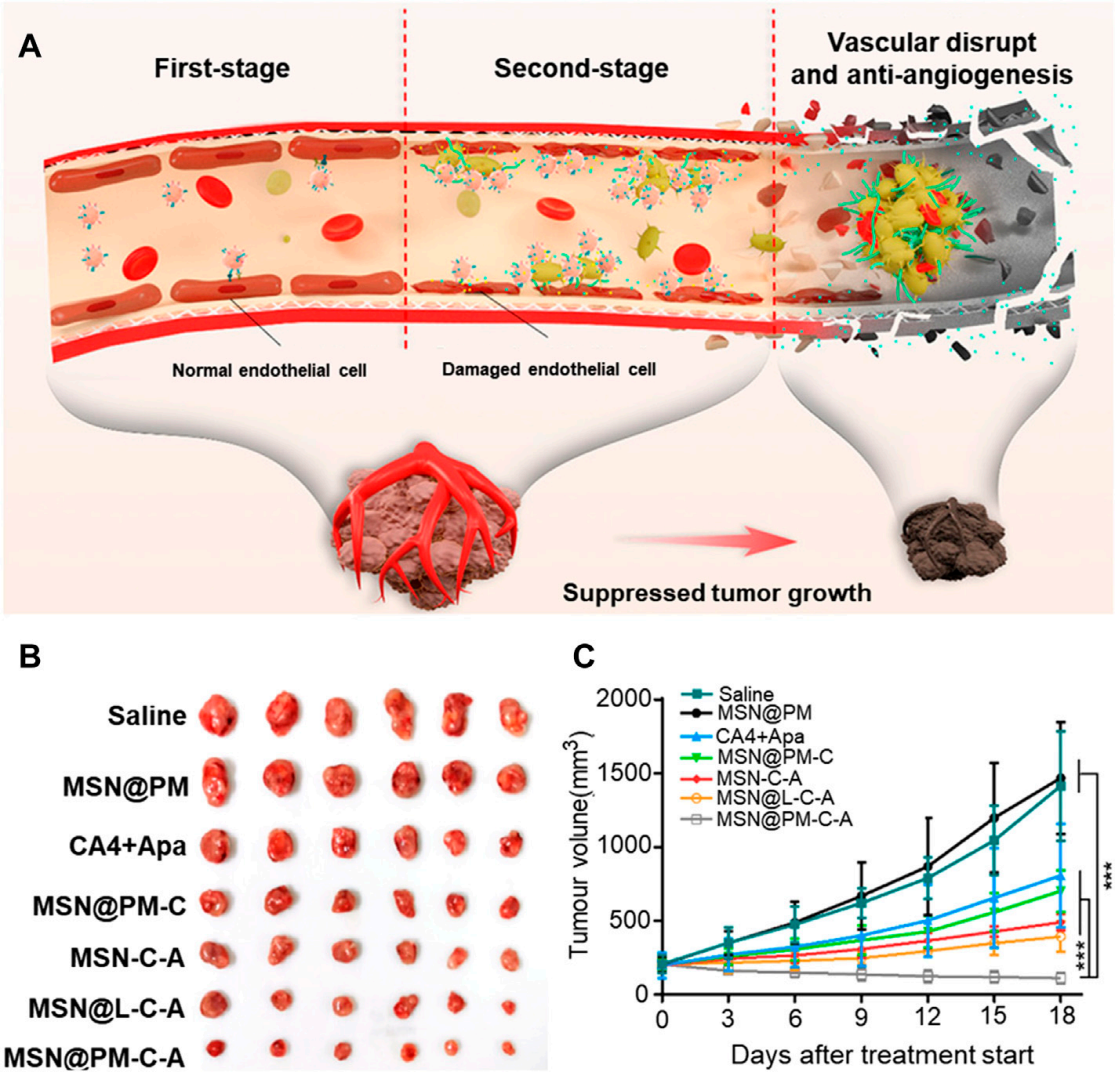
Illustration of the proposed action mechanism and the tumor-killing effect of MSN@PM-C-A in tumor vessels. **(A)** Proposed antitumor mechanism of MSN@PM-C-A in the tumor-bearing mouse model. **(B)** Pictures of mice with treatments. **(C)** Tumor growth curves of MHCC-97H tumor-bearing mice treated with different agents. Reprinted with permission from (Li et al., 2021a).

Studies have shown that platelet-coated nanoparticles can avoid rapid blood clearance and the activation of the immune system, and have better biological properties than liposomes. Li et al. designed a platelet membrane (PM) coated with mesoporous silica nanoparticles (MSN), co-loaded with cobrestatin A4 (CA4) and the antiangiogenic drug apatinib (Apa), for combination therapy (Di Paolo et al., 2013). Nanoparticles coated with the platelet membranes bound to the endothelial cells of the tumor by targeting proteins of receptors on the surface of the membrane to destroy the blood vessels. The damaged blood vessels attracted new nanoparticles to gather, thus realizing the self-amplification and aggregation of nanoparticles at the tumor site (Figure 3A). Thus, active tumor targeting and intra-tumor vascular disruption can be rendered interdependent and mutually reinforcing to yield significant anti-tumor effects. In addition, the two antiangiogenic agents acted on the tumor vessels in different directions to enhance these effects. The results also proved that a combination of the two drugs is more effective than monotherapy. However, this strategy is simply an enhanced version of the starvation therapy, in which tumors were killed without nutrients and oxygen. And it could not prevent the remaining tumor cells from acquiring material from the surrounding normal tissue, allowing the tumor to be inadequately cleared. When combined with conventional therapies that mostly target peripheral tumor cells, the tumor can be better suppressed (Figure 1B,C).

Drzyzga et al. combined DMXAA (5,6-dimethylxanthenone-4-acetic acid) with radiotherapy to suppress tumor cells and the effect of the treatment (Drzyzga et al., 2021) (Anselmo et al., 2014). DMXAA was used to destroy vascular endothelial cells of the tumor to expose collagen and activate platelets, which aggregated into clumps to form thrombus through positive feedback. The infarction agent caused necrosis at the center of the tumor and attracted the immune cells to gather there, but could not suppress the surrounding tumor cells as well (Liu et al., 2017). Radiotherapy was able to compensate for this defect so well that the combination of the two treatments destroyed 50% more of the tumor than when either one of them was used.

Patients often suffer from blood abnormalities, platelet dysfunction, and other diseases, such as abnormal liver function and anemia, that change the number and state of their platelets (Newsome et al., 2018; Bellelli and Tame, 2022). An insufficient number of platelets or dysfunction in them might lead to thrombosis-related disorders (Zhou et al., 2021). Yang et al. designed platelet-like nanoparticles (pNPs) based on self-assembling peptides to stimulate the initiation of blood clotting and the formation of clots in tumorous blood vessels (Yang et al., 2020). pNPs first specifically bound to membrane glycoproteins (e.g., CD105) that were overexpressed on the vascular endothelial cells of the tumor (Nair et al., 2020). They were then activated into platelet-like nanofibers (apNFs) through ligand–receptor interactions. Following this, the apNFs acted as activated platelets to expose more binding sites, and recruited and activated additional pNPs in a similar manner to that in platelet aggregation to form clots (Jurasz et al., 2004). The targeting sequence and self-assembling sequence in the molecule enabled the bionic coagulation to efficiently form a precise fiber network. *In-vivo* and *in-vitro* experiments have shown that drugs can intelligently and accurately construct artificial clots to significantly hinder the growth of tumors. pNP-induced artificial coagulation offers more promise than natural coagulation for treating diseases caused by the dysfunction of the blood vessels related to platelets.

## Conclusion

Novel approaches to treating tumors are driving a growing number of researchers to attend to the treatment of tumor vascular infarction. This article summarized three methods of drug activation that inhibit the growth of the tumor by causing vascular infarction, blocking substances from entering the tumor, and causing its necrosis. However, border cells can absorb nutrients and oxygen from normal tissues, which leads to the recurrence and metastasis of the tumor. This has prompted researchers to use vascular infarction therapy in conjunction with other treatments.

Drzyzga et al. combined vascular infarction therapy and radiotherapy to explore the best delivery conditions to improve treatment (Drzyzga et al., 2021). Thrombin and chemotherapy drug doxorubicin (Dox) have also been integrated into a single nanocarrier to enable chemotherapy and blood coagulation to work together. This is a feasible and reasonable means of attacking the tumor from different directions, and the two treatments complement each other’s advantages to minimize the number of residual tumor cells (Li et al., 2020).

However, the following problems still need to be solved in future work in the area:

(1) High cost: Regardless of whether peptides or nanorobots are used, it is important to ensure that the cost of treatment is acceptably low.
(2) Rapid metabolism of biomaterials: In addition to not producing immune resistance to the human body, biological materials can be degraded in the body without producing harmful substances. However, they are quickly metabolized. Extending their duration of circulation in the body by slowing down their metabolism requires more research.
(3) Inefficient drug delivery: Most current TIBs are based on the EPR effect for tumor targeting and accumulation, and disorder of the blood vessels in the tumor leads to high intravascular pressure such that many drugs cannot be delivered to the tumor. We thus need to develop intelligent biological materials that are responsive to the pH, have redox potential, and are sensitive to overexpressed enzymes in the TME to increase the likelihood of the drugs acting on the tumor.
(4) Biological safety: The number of platelets in tumor patients is higher than in normal people. If the biological material cannot accurately release the blood coagulation drug, thrombus can occur in normal blood circulation and cause organ damage. Therefore, doctors often reduce the amount of medication for patients during treatment, but this limits the use of large doses of drugs and leads to incomplete tumor elimination. Residual tumor cells can then rely on the remaining blood vessels to grow and metastasize.

We have summarized recent progress in research on the vascular obstruction of the tumor from three perspectives: activating the thrombin pathway, activating fibrin, and activating platelets. We concluded that the problems of poor water solubility and non-specific targeting can be solved with the help of biological materials. Coagulation drugs with a short half-life in the body (such as thrombin, coagulase, tissue factor, and DMSSA) can reach the vascular site of the tumor, and can even use biological materials as carriers to combine coagulation therapy with other, complementary treatments (radiotherapy, chemotherapy and neovascularization suppression therapy). This significantly improves the ability to kill tumors and lowers the tumor’s resistance to drugs. Coagulation therapy does not affect cell survival by changing the intra-cellular mechanism, and starves the tumorous cells by blocking the blood vessels so that they cannot alter their mechanism to escape, as they do in response to chemotherapy. In particular, infarction drugs supported by biological materials, whether activated by the thrombin pathway, platelet pathway, or fibrin pathway, are safer for the patient and more accurate at targeting the tumor than single drugs. Tumor vascular infarction therapy thus offers a wide range of options for tumor treatment. The emergence of biological materials has accelerated the emergence of such treatments, although daunting challenges persist to their use in clinical applications.
